# Dose-Response Recovery of Probiotic Strains in Simulated Gastro-Intestinal Passage

**DOI:** 10.3390/microorganisms8010112

**Published:** 2020-01-13

**Authors:** Sofia Forssten, Arthur C. Ouwehand

**Affiliations:** DuPont Nutrition and Biosciences, Sokeritehtaantie 20, FI-02460 Kantvik, Finland; sofia.forssten@dupont.com

**Keywords:** *Lactobacillus*, *Bifidobacterium*, probiotic, simulated digestion, dose-response

## Abstract

The probiotic definition stipulates “adequate amounts”. Here, we investigated the metabolic output and recovery rate of probiotic strains using a simulated upper gastro-intestinal passage and colonic fermentation. Two different doses, 7 × 10^9^ colony forming units (CFU) and 7 × 10^10^ CFU, of a probiotic mixture (*Bifidobacterium lactis* Bl-04, *Lactobacillus acidophilus* La-14, *Lactobacillus paracasei* Lpc-37, and *Lactobacillus plantarum* Lp-115) or placebo were tested. The four strains were quantified by qPCR and the metabolites analyzed by gas chromatography. There was a dose-response in the detection of all four strains. There was a slightly larger increase between the two doses for *L. paracasei* Lpc-37 as compared with the other strains; this may suggest a greater robustness of this strain. Compared with the placebo, the high dose simulations generated more propionic acid and a higher total of short chain fatty acids (SCFA). Higher doses of a species are required to reach measurable increases above the baseline level of this species.

## 1. Introduction

Probiotics are defined as “Live microbes that, when administered in adequate amounts, confer a health benefit on the host” [1]. This definition is valid for both single- and multi-strain probiotic products. Probiotics are often marketed as combinations of strains. The assumption is that a combination of strains leads to improved efficacy, although this is not always clear from the literature [2]. However, we have recently shown that negative interactions should not be expected between strains in a product [3]. What is less documented is whether there is a benefit from using higher doses. A recent systematic review suggested that although this is largely unknown, for antibiotic associated diarrhea there is a clear indication that a higher dose does seem more beneficial [4]. The same systematic review also documented that higher doses do lead to higher fecal recoveries. Higher doses of probiotic mixtures have elsewhere been reported to be associated with earlier, longer, and higher fecal recovery [5]. The present study therefore aimed to investigate in vitro the effect of two different doses of a probiotic mixture on the separate strains’ recovery rate and their metabolites.

## 2. Materials and Methods

### 2.1. Investigated Products

Two different doses, 7 × 10^9^ colony forming unites (CFU) and 7 × 10^10^ CFU, of “Flormidabil” (Sandoz, Origgio, Italy) were tested. The product consists of *Bifidobacterium lactis* Bl-04 (ATCC SD 5219, 75% of total counts), *Lactobacillus acidophilus* La-14 (ATCC SD 5212, 20% of total counts), *Lactobacillus paracasei* Lpc-37 (ATCC SD 5275, 4% of total counts), and *Lactobacillus plantarum* Lp-115 (ATCC SD 5209, 1% of total counts) contained in excipients. The placebo consisted only of the excipients: hydroxypropyl methylcellulose, microcrystalline cellulose, magnesium salts of fatty acids, silicon dioxide, and titanium dioxide, and was free from probiotics.

### 2.2. Simulated Digestion

The digestion was simulated as described earlier [6]. Briefly, two daily servings of probiotic mixtures or placebo were mixed with a phosphate–carbonate buffer, and the pH was adjusted to 6.5 and incubated for 5 min with α-amylase at 37 °C. Subsequently, the pH was adjusted to 2.5 with HCl, pepsin was added, and the mixtures were incubated for 1 h 15 min at 37 °C. This time was chosen to reflect the relatively short gastric residence time of low viscosity foods. The pH was subsequently neutralized to 6.5 with NaOH, and pancreatin, lipase, and bile were added and incubated for 1 h 30 min at 37 °C. Finally, the bacteria were harvested by centrifugation, and the supernatant was removed; the bacteria were subsequently resuspended in a simulated ileal medium [7].

The colonic fermentation of the two different doses of probiotic preparations and the placebo were simulated in four separate experiments using an in vitro model of the human colon. For reference, an unsupplemented control simulation was also run in parallel. The structure and function of the semicontinuous human colon simulator has been previously described [7,8]. Briefly, the simulator consists of eight parallel units, each of which comprises four sequentially connected glass vessels (V1–V4). These eight units can be run simultaneously; therefore, the control (no supplementation in addition to the fecal inoculum), as well as the two probiotic doses (7 × 10^9^ CFU and 7 × 10^10^ CFU) and the placebo (no probiotics) were run in parallel. The conditions (temperature, pH, anaerobiosis) in the simulator vessels are adjusted to represent the different compartments of the human colon: vessel V1 represents the ascending colon, vessel V2 the transverse colon, vessel V3 the descending colon, and vessel V4 the end of the descending colon and the sigmoid/rectum area. The set pH levels in the simulator vessels (5.5, 6.0, 6.5, and 7.0) are similar to those measured in different compartments of the human colon [9] and are adjusted with gaseous ammonia when levels decrease below the target values during simulation. Also, the working volumes in the vessels increase from the proximal to the distal end (3, 5, 7, and 9 mL), mimicking the reducing flow towards the end of the large intestine. The simulator was kept under anaerobic conditions at 37 °C. All run parameters, including pH control, gas, and liquid transitions, were computer regulated using customized software.

Prior to the start of the simulations, a fresh fecal sample was obtained from adult human volunteers. The four donors were apparently healthy adults following a typical Western diet. They had not been on antibiotic therapy during at least the past 3 months and had not been consuming any probiotic products for at least 4 weeks prior to the study. The feces from a single donor were used to run one set of simulations. Fresh fecal material was preconditioned by diluting it with 3 parts (*w/v*) of anaerobic ileal simulator medium and filtered through a 0.3 mm metal mesh, where it was afterwards anaerobically incubated for 24 h at 37 °C. Prior to the inoculation of the simulator with fecal microbes, all tubing and vessels in the simulator were flushed with oxygen-free nitrogen gas for 1 h and filled with autoclaved tap water to preset levels (3, 5, 7, and 9 mL, respectively). Then, 10 mL of anaerobic 0.9% NaCl solution (*w/v*) were fed to the first vessel and mixed before the excess amount was transferred to the next vessel, leaving a volume of 3 mL in the first vessel. The same procedure was repeated for the other vessels. The overnight-conditioned fecal sample from a single donor was used to inoculate the simulator units. After initiation of the simulation, 3 mL of fresh medium including the test substrates was pumped into the simulator system (vessel V1) every 3 h. During the simulation, transitions of the fermented media between the vessels and the feeding of fresh fluids occurred with 3 h intervals. The simulations were continued for 48 h, after which the fecal slurry samples from all vessels were collected. At this point, the volumes in the vessels were 6, 8, 10, and 12 mL [3].

### 2.3. Sample Analysis

Bacterial DNA was extracted from the treatment samples by an initial bead beating step of two 3 × 30 s cycles at 6800 rpm with a Precellys bead beater (Bertin Instruments, Montigny-le-Bretonneux, France), where the DNA was afterwards purified and extracted with an automated MagMAX™ Sample Preparation System (Life Technologies, Halle, Belgium), using the MagMAX™ Nucleic Acid Isolation Kit. The quantity of extracted DNA was determined by a Qubit^®^ dsDNA HS Assay Kit (Thermo Fisher Scientific, Vantaa, Finland). Strain and/or species-specific qPCR were performed for the probiotics from all vessels (V1–V4) of all simulations on 7500FAST real-time PCR instruments (Applied Biosystems, Waltham, MA, USA), using FAST protocols and SYBR or Taqman chemistries [3]. Primer sequences and annealing temperatures are presented in Table 1. The qPCR method does not distinguish between live and dead cells, and the results are therefore expressed as number of gene copies.

To understand the influence of the probiotics on the microbial activity, short chain fatty acids (SCFA) and branched chain fatty acids (BCFA) were determined as described by Ouwehand and coworkers [10].

###  2.4. Statistical Methods

Results from the four separate simulations using inocula from four donors, one donor per simulation, are expressed as mean and standard error of mean (SEM). To facilitate the overview of the data and the comparison between the treatments, the data from the four vessels of each channel were combined for further analysis.

The statistical significance between treatments was determined by a pair-wise Student’s *t*-test. The statistical analyses were blinded.

## 3. Results

**Figure 1:**
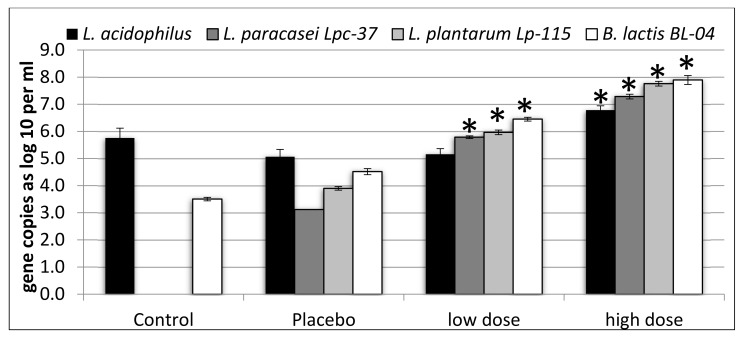
Gene copies of *Lactobacillus acidophilus*, *Lactobacillus paracasei* Lpc-37, *Lactobacillus plantarum* Lp-115, and *Bifidobacterium lactis* Bl-04 per mL of colonic simulation liquid. Results are expressed as mean and standard error of mean from four simulations, each simulation with fecal material from a different donor. Asterisks (*) indicate statistically significant (*p* < 0.05) differences, as compared with both the control and the placebo.

Compared with the control and placebo, the counts of *L. paracasei* Lpc-37, *L. plantarum* Lp-115, and *B. lactis* Bl-04 were significantly higher in the simulations with the low dose (*p* < 0.05). Counts for *L. acidophilus* were not different between low dose simulations and the control or placebo (*p* > 0.05) (Figure 1). Counts of *L. acidophilus*, *L. paracasei* Lpc-37, *L. plantarum* Lp-115, and *B. lactis* Bl-04 were significantly higher in simulations with the high dose, as compared with both the control and the placebo (*p* < 0.05) (Figure 1). When comparing low dose and high dose simulations, *L. paracasei* Lpc-37, *L. plantarum* Lp-115, and *B. lactis* Bl-04 were significantly higher in the simulations with the high dose (*p* < 0.05). For *L. acidophilus*, the difference between the high dose and the low dose simulations was not significant (*p* = 0.0907) (Figure 1). The differences between the low dose and the placebo varied from log10 0.09 (*L. acidophilus*) to 2.67 (*L. paracasei* Lpc-37), while the differences between the high dose and the placebo varied from log10 1.72 (*L. acidophilus*) to 4.17 (*L. paracasei* Lpc-37). The difference between the two doses for the four organisms varied between log10 1.44 (*B. lactis* Bl-04) and 1.79 (*L. plantarum* Lp-115).

**Figure 2:**
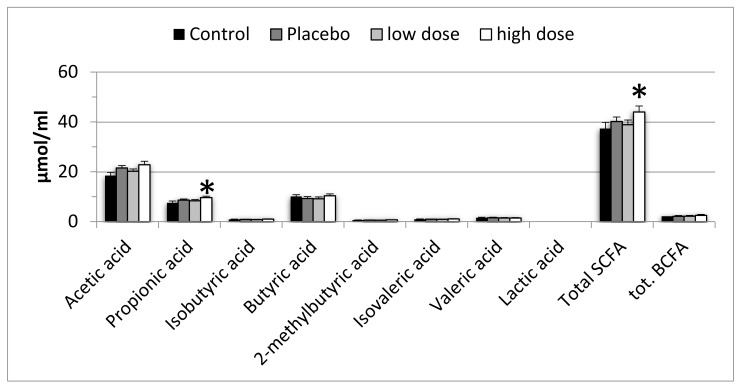
Short chain fatty acids (SCFA) and branched chain fatty acids (BCFA). Results are expressed as mean and standard error of mean from four simulations, each simulation with fecal material from a different donor. Asterisks (*) indicate statistically significant (*p* < 0.05) differences, as compared with both the control and the placebo.

Few differences were observed in fecal metabolites. Although the differences were small, the high dose simulations, when compared with the control, had more propionic acid (*p* = 0.0031) and more total SCFA (*p* = 0.0110). No other differences were observed (Figure 2).

## 4. Discussion

The probiotic definition refers to the administration of an “adequate amount” [1]. What this amount is not further defined, as it depends on the probiotic (combination) and the health target. In general, the dose clinically documented to provide a health benefit should be taken as the minimum amount. There is, however, a tendency in the market to interpret this as meaning that a higher dose is better [15]. While the evidence for this general conclusion is limited and would benefit from further research, it appears that for antibiotic-associated diarrhea, higher doses are more efficacious [4]. Further, the recovery of higher levels of fed probiotics from feces typically results from the consumption of higher doses [4,5]. In addition to higher counts, there is also a marketing trend to include a higher number of strains in probiotic formulations [16]. There are indications that in some cases multi-strain formulations may be more beneficial than single-strain products, but the evidence is, again, limited [2]. We have shown earlier, in simulated digestion experiments, that in multi-strain formulations strains do not negatively impact each other as compared to the single strains [3]. The aim of the current study was to determine to what extent strains in a multi-strain probiotic formulation at two different doses survive simulated digestion. Further, we assessed the impact of the doses on metabolite production.

The simulations had a relatively high background level of endogenous *L. acidophilus*. They were nevertheless within the range of 10^4^–10^7^ gene copies per gram, as we have observed before [3,17]. This explains why the low dose did not increase the levels of *L. acidophilus* as compared to the control and the placebo. In earlier simulations, the higher tested doses of *L. acidophilus* NCFM were also observed to increase *L. acidophilus* levels [3,17,18]. This thus suggests that higher probiotic doses are required to increase fecal levels of a particular species above the background. All three other microbes were significantly increased in their levels with both low and high dose simulations, which is in agreement with earlier observations [3,17]. The increase in the counts was dose dependent, with the counts in the higher dose simulations being more than one log10 higher than in the lower dose simulations, in agreement with a recent human intervention study on the same probiotic product [5].

Although *B. lactis* Bl-04 formed the largest part of the product (75%), it did not generate the largest increase in counts, as compared to the placebo. This is in contrast to a recent human study with the same probiotic products, where *B. lactis* Bl-04 reached the highest fecal recovery of the four strains [5]. The biggest increase in counts as compared to the placebo was caused by *L. paracasei* Lpc-37 in both doses tested, even though this strain was only a minor component in the product (4%). This may indicate a difference in the simulated GI-survival of the strains included in the product. The difference between the two doses for each of the microbes ranged from log10 1.44 to 1.79, which is only slightly more than the difference in concentration between the two products, 1 × Log10. The strains thus survived the simulated digestion very similarly, regardless of the dose. There may thus be a difference in the survival of simulated digestion, with *L. paracasei* Lpc-37 being more robust than the other tested strains [19]. However, there may also be technological reasons for the higher relative recovery of *L. paracasei* Lpc-37, such as a difference in DNA recovery efficiency between the strains and the subsequent PCR efficiency [20,21].

Although a significantly higher level of propionic acid was detected in the high dose simulations, the difference is small, especially when compared with the effect fibers have on the type and amount of SCFA [22]. Also, the total amount of SCFA was increased by the high dose probiotics. This increase is also modest, but interestingly driven by apparent increases in several other SCFA, in addition to propionate. Nevertheless, the biological relevance of this modest increase remains to be determined.

For further understanding of the dose-response effects of probiotics, it would be relevant to investigate the relation between cell number and adhesion in various models, such as Caco-2 and HT-29 cells or intestinal mucus. Earlier work has shown adhesion to mucus to be linear for probiotics but asymptotic for probiotic adhesion to Caco-2 cells for concentrations of up to 4 × 10^8^ CFU/mL [23]. In addition to differences in survival and detection, differences in adhesion may also play a role in the detected levels of probiotics in vivo [5].

## 5. Conclusions

The strains survive the simulated gastro-intestinal passage in a clear dose-dependent manner, where a ten times higher probiotic dose results in a slightly more than ten times higher recovery. The results also show that higher doses may be needed to increase the counts of a particular species above high background levels. Further, the results show that the higher dose, in contrast to the lower dose tested, may influence microbial metabolism.

## Figures and Tables

**Table 1:** Primer sequences and annealing temperatures.

Species/Strain	Primer Name	Sequence	Anneal Temp. [°C]	Reference
*B. lactis* Bl-04	Bl04_for	CTTCCCAGAAGGCCGGGT	60	[11]
	Bl04_rev	CGAGGCCACGGTGCTCATATAGA		
*L. acidophilus*	Laci_NCFMMJ_RTfwd	CCACGACCAGATGTAACCAA	62	[12]
	Laci_NCFM_Rtrev	TTAGAAGATGCCAACGTCGAG		
	Laci_NCFM_probe	5’HEX TAA GCC GAA-ZEN-CAA TGC TGA AAC GAT 3’IABkFQ		
*L. paracasei* Lpc-37	F_paca_IS	ACATCAGTGTATTGCTTGTCAGTGAATAC	60	[13]
	R_paca_IS	CCTGCGGGTACTGAGATGTTTC		
	P_paca_IS	5’ FAM TGCCGCCGGCCAG 3’ IBQ		
*L. plantarum* Lp-115	LP115_F	CTTGATGACTCTTCTGGGGC	60	[14]
	LP115_R	ACGGGAGTGATAGACGTTGAG		
	LP115_P	TTGAGTGCAGCGTTGTTTGCGAGCGTCC		
